# Animals and Medicine

**Published:** 1985-07

**Authors:** I. A. Silver

**Affiliations:** Professor of Comparative Pathology, University of Bristol


### Animals and Medicine
### The Long Fox Memorial Lecture

**I. A. SILVER**
Professor of Comparative Pathology, University of Bristol

It is customary to begin this lecture with a brief reminder of the life and achievement of Edward Long Fox who came from a Bristol family of distinguished medical men. After a wide ranging education taking in Shrewsbury, Edinburgh, London and Oxford he returned to Bristol where he worked at the Royal Infirmary for most of his career. He was a modest man of great charm and intelligence, prominent among those who, in the nineteenth century, formed a tightly knit band of acute observers of human behaviour in health and disease. He made significant contributions to the understanding of a number of infectious diseases and was in the forefront of rational medical thinking, a position that was not necessarily comfortable and sometimes drew him into conflict with his more traditionally minded colleagues. This originality of thought was particularly to his credit in that he was the product of a teaching system that still retained great faith in 'authority' and which even then was shackled to some extent by the opinions of the sages of antiquity and the Middle Ages. Progress in medicine was still relatively slow but starting to accelerate and the fundamentals of many disease processes were only beginning to be understood, or remained completely obscure and provided grounds for endless, and often fruitless argument. The experimental approach to medicine was in its infancy and largely confined to Physiology and Bacteriology. Edward Long Fox was an enthusiastic proponent of Koch's views on the microbial origin of tuberculosis. He used these enlightened ideas on the spread of disease in the successful control of outbreaks of typhus and cholera in Bristol.

We do not know if the comparative approach to Medicine interested him but in his day there was not only an intellectual but also a social divide between human and animal medicine which was reinforced by the separation of the medical and veterinary professions into those who attended at the front door and those who usually went to the back of the house. However, he lived on to see the early phases of the impact of scientific investigation in medicine. Sadly he became a victim of diabetes before the basis of that disease has been demonstrated by the experimental observations of Houssay and others on dogs and died before the work of Banting and Best revolutionised the survival prospects and quality of life for diabetics. While great strides were being made by microbiologists in the latter part of the last century in understanding the nature of bacterial disease in man and animals, the situation regarding viral diseases was less clear. It was only in 1898 that foot-and-mouth disease of ruminants was shown unequivocally to be caused by a 'filterable virus'; the first such demonstration of a virus disease in mammals as opposed to the classic work in plants on tobacco mosaic virus. However, Long Fox and several of his contemporaries had suspected the existence of minute infective particles for several decades, while the likely role of such agents in human disease had been a topic of lively debate in his grandfather's day, many years before the existence of micro-organisms had been indisputably demonstrated by Pasteur and Koch.

The rapid increase in medical knowledge and the consquent improvement in human and animal health that has occurred in the past century has been brought about largely by the interaction of physicians, scientists and veterinarians and the increasingly rapid transfer of information gained on one species to the benefit of others. Until recently there was a great reluctance among some members of the medical profession to accept that ideas or practices produced by the veterinary profession were anything more than a pale reflection of their own achievements: a view often reciprocated in reverse by the veterinarians. However, this destructive divide between the two groups was of relatively brief duration and is fortunately fading away.

We know that in Xerxes' army there were both physicians and veterinarians and it seems that their roles were to some extent interchangeable. No Royal College examinations or E.E.C. directives confined practitioners to a particular species. This happy freedom from regulation did however carry a certain penalty with it, for it is recorded that Xerxes, being displeased with the attentions of his primary medical advisors, disposed of them and turned to his chief horse doctor for treatment. Apparently he obtained relief from his symptoms and was generous in his initial reaction. Unfortunately for the horse doctor, Xerxes' malady recurred and the disillusioned emperor crucified the veterinary officer presumably on the grounds of personal pique or possibly through invoking a contemporary equivalent of the Trade Descriptions Act. Xerxes' reaction may seem a little excessive to modern tastes but similar treatment of the medical or veterinary professions today might be welcomed by the present government and would certainly banish the spectre of the enforced reduction of student intake to medical and veterinary schools with which we are faced.

Having as it were, set the scene in relation to the life and times of Edward Long Fox, I will attempt to give a brief historical perspective of the relationship of animals to medicine and then continue with an evaluation of that association as it exists today. In antiquity, or rather in those areas of the world that recorded their doings in antiquity, we know that the status of animals ranged from venerated god to unconsidered chattel. Animals might be prized as magical agents for the cure or avoidance of certain diseases but could equally readily become the victims of sacrifices or the ingredients of medicinal products.

A very early feature of the relationship between man, animals and medicine was the idea that in some way the 'virtue' of an animal as conceived at the time, could be passed on to a human recipient in the form of sympathetic magic or medicine derived from the unfortunate creature. Such a belief, badly stated, may seem difficult for us to accept, yet we happily derive immunologically active products from contemporary animals in the almost unquestioned certainty that they will protect us from specific diseases such as tetanus or diphtheria. Nevertheless we feel that we have progressed to some extent in that we no longer approve of or generally believe in boiling live puppies or baking live storks to produce magic potions that cure all ills, while 'eye of newt and toe of frog' are not regularly available on the National Health Service, however much the latter may be derided. 'Liver of blaspheming Jew' has of course been barred under the Race Relations Act but was probably going out of fashion even before the nineteen seventies. In the category of animal related medical magic we can probably include the killing and eating of traditionally 'courageous' animals such as polar bears (or ferocious human foes) which were reputed to pass on their desirable psychological characteristics to the consumer. A word of caution here to those tempted to try ancient prescriptions; the eating of polar bears may provide strength and courage but modern mythology insists that consumption of the liver leads rapidly to Vitamin A intoxication, while dining on the brain of a valiant human animal may result in slow death from Kuru or from Creuzfeldt–Jacob disease.

The most intriguing survival of sympathetic magic in the 20th century is rooted in the reluctance of most human males (and some females) to accept the inevitable decline in their sexual prowess with advancing years. The poaching of rhinoceros horns and the sale of antler velvet bears witness to the continuing market in supposed aphrodisiacs. The phallic outline of the rhino horn and the insatiable lust of the rutting stag still provide hope of restoration of failing youth to many elderly or impotent men in the Far East, and probably nearer home as well.

In the Middle Ages great store was set by the teachings of the Egyptian, Greek and Roman physicians of the Classical Period, much of which was based on sound observation and inductive reasoning. Unfortunately it also contained many misconceptions about the nature and cure of disease, and to these were added the rather less rigorously tested beliefs derived from the Old Religion, and the worship of the Earth Mother. The supposed properties of the Bezoar Stone provide a case in point. It was generally believed that the common bovine enterolith (bezoar) protected cattle, and by extension would also protect people, against the effects of poison. This was a matter of great interest to monarchs who, throughout history, have been in constant fear of poisoning by their friends, family or rivals. King James VIth of Scotland (and Ist of England), despite his fear of witchcraft, was a keen observer and only inclined to accept folklore if it suited his purposes. He therefore decided to test the efficacy of the Bezoar in what must have been one of the first controlled medical field trials of a biological agent. He obtained volunteers from among condemned prisoners in London by offering them their freedom if they survived the administration of poison by the Court Physician, after having been given a 'protective' dose of Bezoar Stone. It says much for their desperation or their faith in the Stone that there was no lack of volunteers. It is also clear that the Court Physician took a rather relaxed view of his Hippocratic oath because he gave the unfortunate prisoners mercuric chloride which produced equally dramatic and horrifying results in those who had received the Bezoar and on those who had not. Thus perished not only a long standing myth but several people, in what might be described as an early $LD_{100}$ test.

Folk medicine was inextricably interwoven with magic and ranged in its use of animals from the whimsical to the malign. Even in this century, certainly in my childhood and perhaps until today, the swallowing of a whole mouse was widely believed to be a specific cure for whooping cough. In some districts the mouse was fried but in others this was regarded as unnecessary and probably 'Un-British'. Apart from introducing the likelihood of salmonella infection and the possibility of Weil's disease, the procedure was probably no more harmful, except to the mice, than adminstration of Pertussis vaccine.

Some rather less praiseworthy activity was centred around the reputed medicinal qualities of toads. These amphibians are of course well protected against most natural predators by the possession of a very virulent poison (Bufotoxin) in the skin which, together with their wrinkled appearance and startlingly beautiful bright eyes gave them an evil reputation. While noble maidens from impoverished backgrounds were well advised to try kissing the odd frog in the hope of turning him back into a rich and handsome prince, it is to be hoped they were warned against similar affectionate gestures towards toads. Even if the toad was not an emissary of the Devil, as was generally believed, the powerful neurotoxin in its skin can be absorbed easily through the lips and cause blockage of the $Na^+$ channels of electrically active cells; an experience considerably more unpleasant than some that have been classified as worse than death. The medicinal uses of toads ranged from straight homicide to the establishment of psychological dominance over other people or animals. This latter was achieved by capturing a grey marsh toad at full moon, hanging it in a thorn bush until it was dead, burying it in an ant hill until the next full moon and taking its bones to a running stream. When these were placed in the water all would sink or float down stream except one which was the special 'toad's bone'. Possession of this talisman gave the owner power over all animals and sometimes people, but there was a Faustian penalty to pay at the end of the day. Even in the 1880s a pamphlet published for stockmen contained instructions and warnings about the use of the Toad's Bone, among many similar gems of arcane information.

One of the animals most closely associated with medicine and even bearing that association in its proper name, is the leech (Hirudo medicinalis) which in return bestowed its own name on the medical and veterinary professions. Much beloved of physicians and 'horse doctors' in the days of 'bleeding', leeches suffered a sharp decline in popularity when blood-letting went out of fashion as a panacea but they are now making a comeback in orthodox medicine for the treatment of certain specific conditions such as polycythaemia and haematomata. Perhaps their renewed use will be stigmatised as 'exploitation' by some proponents of Animal Rights but leeches, with Dracula and Vampire bats, know what they like.

Our relationships with the Invertebrates have not usually been so happy and it is reasonable to make use of this association to form a bridge from mediaeval to modern medicine. Many of the major scourges of mankind have either been caused by parasitic animals or carried by them. The elucidation by Ross and his contemporaries of the biology of the malarial parasite and its association with Anophylene mosquitos forms one of the great feats of detection, as do the stories of trachoma and Simulids or of Trypanosomiasis and Glossina or Rhodnius. Long Fox himself was involved in the control of typhus in Bristol and was acutely aware of the role of the louse in its spread.

While the dangers posed by these pathogenic creatures and their vectors have been greatly diminished in countries with a high standard of living and of Public Health legislation they reappear with uncanny precision wherever there is a breakdown of civilised living. As one of the fortunate nations we have long been relatively free of most of the major infectious and parasitic diseases although it should be remembered that the 'ague' of marshy areas in Britain was malaria which re-established itself temporarily in East Anglia after the first World War. Our priviledged position has been due in part to the efforts of enlightened physicians such as Long Fox, in part to our civil engineers, in part to education and improved personal hygiene, in part to the greater understanding of these diseases gained from experimental medicine and in part to our ruthless suppression of those animals that annoyed, endangered or competed with us. We have come to regard such things as freedom from lice, ticks and fleas, from tape worms and round worms or from scabies and malarial mosquitoes as entirely natural. We do not expect to have to dodge wolves or escape from ravenous bears on our way to work and we are outraged if our food is infested with parasitic worms or if we have to share our beds and houses with bedbugs or rats. Yet this happy state of affairs is entirely artificial as anyone who has lived in the less developed parts of the world, worked in slum areas or read any medical history, will recognise.

In recent years there has been an increasing awareness of our exploitation of animals in many fields and a heightened interest in the ethical question of man's right to use animals for his own benefit. Criticism of biomedical science by the Animal Rights movement has mostly been directed against the use of animals for making new discoveries or for safety testing of medicinal or household products. While a greater awareness of animal welfare needs can do nothing but good, absolute condemnation of the use of animals in research would not only halt progress in human but also in veterinary medicine. Animals have been at least equal beneficiaries with man of the results of biomedical investigations, and in some fields, such as nutrition and parasitology have been clear winners. Since new ideas concerning the 'rights' of animals are being propounded in both public and academic debate it seems reasonable to consider the consequences of following some of the more extreme suggestions that have been put forward recently. We appear at first sight to be asked to believe that the lives of *all* animals are sacrosanct and may not be imperilled or disturbed in any way by human needs. It also seems necessary to equate perception of the environment by animals with that enjoyed by man. In some cases religious tenets have been invoked in the cause of animal rights without any acknowledgement of the paradox presented by the almost total indifference to animal suffering that is a characteristic to this day of the countries of the Middle East where three of the great world religions originated. The more extreme proponents of animal welfare have been encouraged by a school of philosophy that teaches that *all* animals have rights strictly comparable to the civil rights of the Western democracies and stigmatises traditional attitudes to animals as similar to those that permitted the Slave Trade to flourish well into the last century. An unacknowledged problem with this argument lies in the use of the all-embracing word 'animal'. What is really being discussed in most cases is a very limited range of mammals and to a lesser extent, birds. 'Animals' are usually equated with creatures that can be visualised in anthropomorphic terms. To qualify for 'rights' an animal should be easily visible, clean, furry, and preferably appear in the role of hero in childrens' story books. It is a distinct advantage if it has big eyes and a short face, thus closely approximating to the psychological trigger provided by a human baby's face. It is better still if the audience never has, and preferably never will, actually come into contact with these animals in their natural or even domesticated state. Many illusions about animals have been shattered by looking after a few hens or rescuing an injured piglet from the murderous attentions of its brothers and sisters.

It is a common human characteristic that nothing enables us to hold strong convictions as surely as total ignorance of the subject, whether it be politics, personalities, religion or animals. Regardless of one's view of the tenability of the theses of the more strident members of the animal rights groups, it is noticeable that when presenting their ideas in public, these people do not spend a great deal of time scratching themselves, nor do they give the appearance of suffering from infestations with parasitic worms. Are they not by their lifestyle or medication depriving some needy nematode, or famished flea of home and a meal? Do they tolerate rats and mice in their larders or encourage flies to lay their eggs on the family food? If, as they assert, their philosophy has universal application, why in practice is sympathy and support confined to one small group of mammals and not equally valid for all other animal species, with more legs or none, however unattractive they may seem to human eyes? It has been proposed that 'speciesism' is equally to be condemned as 'racism', which may be true, but if it is to be eliminated the logical consequences must be faced. Perhaps we may yet see a Bed-bug Preservation Movement or a Save the Schistosome Society, but I would be willing to wager a substantial bet on their members being derived exclusively from those parts of the world where bed-bugs are only found in jokes and where no-one is really quite sure whether Bilhartzia is the name of a country, its dictator or a Spanish restaurant.

To many, the animal liberation movement has all the attributes of a moral and political crusade and there can be few indeed who do not sympathise with many of its aspirations. Unfortunately its main aims are not compatible with those of another and possibly larger group who demand total safety in the environment, increase in the quality of life and the elimination of human and animal disease as far as that is practicable. On the one hand there is insistence on the prevention of the use of animals in experiments or test procedures and on the other a demand for absolute assurance that all substances, and especially drugs should be guaranteed as being completely safe for use. Clearly these requirements are not compatible. It is simply naïve to pretend that all drug testing can be done on tissue culture or through computer simulation. Who would care to be anaesthetised by a new formulation that had been tried out solely on a nerve cell culture or to have a new antibiotic that had only been tested in a computer model, administered to their own baby? Nevertheless, the modern Animal Welfare Movement has done a great deal of good in forcing us to examine more critically our attitudes to and exploitation of, other species and to highlight some of the inconsistencies in Society.

There is a popular suggestion that man has brought his ills on himself by abandoning natural life and therfore has no right to demand that animals be sacrificed to extract him from a morass of his making. This idea has some attractions until one considers what is the condition of people in those parts of the world that do not enjoy the benefits of high civilisation. It is immediately obvious that the population in less advanced regions suffer from a much wider range of diseases than we do in our artificial surroundings, in spite of their supposedly blameless existence. Furthermore this philosophy if followed to its limit would appear to deny the benefit of modern orthopaedic surgery, which is derived from and tested on animals, to anyone who is run over by a bus, since someone living the 'natural' life would not be exposed to such a hazard.

In this country animals are better protected from exploitation than anywhere in the world and in several aspects even than our own children. In their relation to Biomedical science they have been the subject of legislation since 1876 when British Veterinarians were so horrified by the cruelty then routinely practised in France in medical training and investigations, that they succeeded in persuading the government to control experiments on animals. The

1876 Act is by no means perfect and is rightly being replaced, although it has served a useful purpose. Those who wish to see the prohibition of all experiments involving animals in this country would be well advised to consider the consequences of such a move. Essential experimental work would have to be transferred to and continue in other countires where animals might not have the benefit of any legal safeguards. While campaigners in the U.K. might feel a glow of righteousness the animals themselves could be considerably disadvantaged.

Among the many medical problems that have beset mankind in the natural world has been the bubonic plague caused by *Yersinia pestis*, usually arising in the Orient and then spreading westwards across the world. This disease is carried by the black or ship rat and transmitted to man by the rat flea *Xenopsylla cheopis*. The influence of the plague on the development of civilisation has been profound and was one of the limiting factors in the growth of cities, and a major cause of changes in social structure in the countryside. It is still a fearsome disease but now that it is better understood, can be combated by elimination of the vectors. A natural limit on its spread seems to have occurred by a mutation in the 16th to 17th centuries when a variant of the plague organism (*Yersinia pseudotuberculosis*) appeared in rodents throughout northern Europe. This bacillus, which causes a devastating disease in many birds and small mammals, does not normally infect man, but the disease protects rats against plague. The spread of this organism in rats thus limited the number able to carry the true plague and therefore reduced the risk to man. This is an example of a natural change which benefited man. However, there is a much better known local story in which the observation of natural infection of man by an animal virus with subsequent development of immunity to a human pathogen has led to world wide artificial application of the process and the disappearance of smallpox from the Earth. Edward Jenner of Berkeley, followed up the observations of Benjamin Jesty 20 years earlier, that milkmaids on his farm who had been infected with cow-pox from their contact with infected cows, were immune to attack by smallpox. Jesty incidently 'vaccinated' his wife and children, while Jenner later introduced vaccination to the general public against widespread opposition. His use of an animal-adapted highly immunogenic virus conferred almost complete immunity against the associated disease in man and was the forerunner of many immunisation procedures which are used today. What he could not have foreseen is the very recent use of the immunogenic part of 'his' virus in the production by genetic engineering techniques of artificial vaccines that confer immunity against other viruses which do not stimulate a very reliable response in their own right.

In the application of immunisation it is difficult to know whether man or animals have gained the most. Before the commercial production of antisera and vaccines, a very high proportion of sheep died each year from a variety of bacterial diseases and thousands of cattle died from lung parasites and various forms of contagious penumonia. Pigs were infected by the hundreds of thousands by swine fever and among domestic pets almost half of the young dog population succumbed to the distemper virus, to leptospiral diseases and to viral hepatitis. In the case of chickens we have reliable statistics of mortality before and after the introduction of modern vaccination. Previously there was a routine expectation of 30 to 40% death rate among young chicks but this has now been reduced to 0.5 to 1% with a consequent reduction in suffering. Virtually all modern domestic animals lead healtheir lives than their predecessors as the result of protection afforded by artificially stimulated immune responses. These advances have flowed from the work of Jenner but have been made possible by experiments on animals, although not necessarily on the species whose protection was being sought. For instance distemper vaccination for dogs mostly stems from investigations on laboratory ferrets, and this work also played a considerable role in the development of a measles vaccine for children which has saved and is saving many tens of thousands of human lives, especially in third world countries. The lack of supplies of this vaccine has contributed to the high death rate among children in the famine stricken areas of Africa.

In a similar vein, discoveries about the nature of herpes viruses in chickens and the application of that knowledge to the development of effective vaccines against those viruses has been of great importance in the elucidation of the behaviour of human herpes viruses and the production of vaccines which it is hoped will control some of them.

So far we have considered the benefits that have accrued to man and animals by what might be termed applied investigation that had a definite practical end in view. However, it is probably not unfair to say that most of the greatest advances in medical science which have produced enormous benefits to man and animals have been derived from experiments on animals where the investigator was undertaking pure research to satisfy his intellectual curiosity, and without any intention or expectation that the work would have any immediate practical relevance. Perhaps the best known examples of this type of work were the investigations that led to the discovery of insulin and which were referred to in relation to the death of Edward Long Fox. The original studies were carried out to investigate the exocrine secretory functions of the pancreas as an exercise in scientific discovery. It so happened that in the course of this work a chance observation led to the conclusion that the pancreas was somehow involved in the control of blood sugar levels and therefore likely to be important in diabetes. This finding triggered an immense amount of work that eventually identified the origins and actions of insulin and ultimately led to its isolation and synthesis. These investigations were carried out on a great many dogs since that species is particularly susceptible to the disease both experimentally and naturally. The benefits to man that have flowed from the work on dogs have been immeasurable and Joslin has proposed that every diabetic child should have a dog to care for and love in partial repayment of the great debt that all diabetics owe to that species. Of course dogs themselves have also benefited from the discovery of insulin since it is used in veterinary practice to control canine diabetes. However, despite the fact that the original discoveries about diabetes were made well over 50 years ago and research has continued on the disease ever since, there is still a great deal we do not understand about it, and therefore features of it that we cannot control, especially in so far as it affects the eyes and the peripheral nervous system. It was this latter complication of diabetes that afflicted Long Fox in his later years.

Some 'anti-scientific' groups have advanced the proposition that we already know enough, or perhaps even too much, to be good for us, so why do any more research? This view is easily countered in the case of diabetes, but it is in any event a council of despair in that it could have been applied at any time in human history. Did we really need the wheel, what is the point of having electricity, and should we have investigated ways of combating cholera, typhoid and poliomyelitis?

The discovery of antibiotics and their subsequent application to the saving of human and animal life shares many of the features of the story of insulin. It derived from a pure research project which had no apparent practical application, yet it revolutionised human and veterinary medicine. The development of these agents involved the use of very large numbers of animals and on that ground has been condemned by some animal rights protesters. Nevertheless, it is difficult to conceive now of the situation before antibiotics were available. Many diseases we now consider trivial, if we consider them at all, were either life threatening or left the victim with a permanent disability, and much modern surgery would be impossible. Those who advocate abandonment of the use of animals in medical research should consider very carefully what such a step would mean to the health of the world, even if they themselves are prepared to live (and die) under the conditions that prevailed in medicine in the 18th and 19th centuries.

The major reason that many of us are alive today, and that most if not all of us are able to enjoy good health, is due to the advances in medicine that have occurred since the days of Edward Long Fox. Most of these developments have been achieved through the use of experimental animals in pure and applied research. It is incumbent upon us not to deny our children the benefit of similar advances, but it is equally important that we recognise our debt to our animals by humane treatment and the use of absolutely minimum numbers, and paradoxically to develop ways of replacing them in the laboratory wherever possible by non-sentient systems.

